# Use of primary health care services prior to suicide in the Norwegian population 2006–2015

**DOI:** 10.1186/s12913-018-3419-9

**Published:** 2018-08-08

**Authors:** Lars Johan Hauge, Kim Stene-Larsen, Tine Kristin Grimholt, Carine Øien-Ødegaard, Anne Reneflot

**Affiliations:** 1 Division of Mental and Physical Health, Norwegian Institute of Public Health, PO Box 222, Skøyen, 0213 Oslo, Norway; 2 Department of Acute Medicine, Oslo University Hospital, Oslo, Norway; 3 Department of General Practice, Institute of Health and Society, University of Oslo, Oslo, Norway

**Keywords:** Primary health care, Mental health, Suicide, General practice, Population-based

## Abstract

**Background:**

Studies report high rates of contact with general practitioners (GPs) in primary care in the time leading up to suicide, particularly among individuals with a history of mental health contact. However, the near lack of studies including population representative controls have prevented investigations into how the contact patterns of suicide victims compares to those of the general population.

**Methods:**

By linking data from two national registries, this study investigated primary health care use in suicide victims aged 15 years and older during the period from 2007 to 2015 (*n* = 4926). Their rates of contact one year and one month prior to suicide were compared to the average rates in the general Norwegian population during the period by estimating relative risks across sex and age. Contact patterns one month prior to suicide were also investigated according to prior mental health consultations in primary care.

**Results:**

The findings revealed a stable trend in contact with GPs in primary care during the observation period, with 79.6% of male and 89.0% of female suicide victims having consulted their GP within a year of the suicide. Corresponding rates one month prior to the suicide were 34.8 and 46.4%, respectively. At both points in time and across all age groups, suicide victims were considerably more likely to consult their GP than were the general population. Suicide victims without prior mental health contact were only modestly more likely to consult their GP within a month of the suicide as compared to the general population, while both the general population and suicide victims with prior mental health consultations had rates of contact well above those without, evident for both sexes.

**Conclusions:**

Contact with GPs in primary care prior to suicide is common in both sexes and across most age groups, in particular for victims with prior mental health consultations. Younger males show the overall lowest rates of contact, and increased alternative efforts to reach this group, in addition to larger population strategies, may pose the most prominent preventive measures.

## Background

Suicide is a serious public health problem and the second leading cause of death among 15–29 year olds worldwide [1]. More than 800,000 lives are lost annually due to suicide, and identifying areas for improved and more effective suicide prevention is therefore of great importance [2]. The period culminating with an individual committing suicide is often one of great interpersonal stress in which help-seeking behaviour is common. The primary health care services is the most readily accessible means of health care available, and with overall high rates of contact on a regular basis, general practitioners (GPs) in primary care may play a prominent role in suicide prevention [3].

Approximately 80% of suicide victims consult GPs in primary care within the final year of the suicide, and about 40% make contact within the last month [4, 5]. Studies investigating primary health care use prior to suicide report higher rates of contact among women than among men [6, 7]. Higher rates of contact are also found among those of older age, while younger individuals, in particular men, tend to show the overall lowest rates of contact prior to the suicide [8, 9]. In addition to higher rates in general, women are also more likely to have mental health consultations than are men [9, 10]. Furthermore, suicide victims with prior mental health contact have higher rates of contact and have consultations closer in time to death than have victims without such contact [11–13]. A considerable share of those with mental health contact prior to the suicide have this contact within a primary care setting [14].

In order to strengthen GPs possibility to recognise contact patterns among individuals ending up taking their own lives, it is vital to be able to contrast their patterns with those of the population at large. Unfortunately, only few studies have included population-representative controls and the majority of such studies have been small in scale [4]. Thus, the extent to which suicide victims’ contact patterns deviates from those of the general population remains largely unknown. Moreover, as there is a more or less complete lack of information about rates of contact for middle age groups, limited knowledge exist on how contact patterns with GPs in primary care may deviate across age. By applying two national population registries, this study will investigate trends in contact with GPs in primary care among suicide victims in the period from 2007 to 2015, and investigate how their rates of contact across sex and age compares to those of the general Norwegian population.

## Methods

### Data sources and study population

The current study investigates primary health care use in the Norwegian population in the period from January 1st 2006 to December 31st 2015. By means of the unique personal identifier assigned to all Norwegian residents, we linked individual data on suicide death from the Norwegian Cause of Death Registry [15] to the national database for the reimbursement of health expenses (KUHR) for all individuals aged 15 years and older. The Regional Committee for Medical and Health Research Ethics granted approval for the study, and both registry owners approved their data to be utilised.

The KUHR database contain data on claims for reimbursement of fee-for-service, among other from GPs in primary care as used in this study. Each registration contain the date of contact, tariff codes reflecting type of service provided, and one or more diagnosis codes according to the International Classification of Primary Care (ICPC-2) [16]. For this study, only tariff codes reflecting a face-to-face consultation at the GPs office were included.

From the Norwegian Cause of Death Registry we included all suicides for whom a minimum of one year of registrations in the KUHR database were possible. All suicides among individuals aged 15 years and older during the period from January 1st 2007 to December 31st 2015 (*n* = 4926) were included, constituting 99.6% of all suicides in Norway in the period [17]. The registry records the date of death and code suicides according to the International Classification of Diseases, 10th revision, during the period (ICD-10 codes X60-X84, Y87.0) [18]. The majority of included suicides were among men (71%), and median age was 46 years for men and 48 years for women.

### Study variables

Using the total number of suicides among men and women aged 15 years and older as respective denominators, we generated rates of contact with GPs in primary care within a year of and within a month of the suicide. In addition to the above, reflecting consultations for any reason, we calculated the rate of individuals with mental health contact in primary care within the year of the suicide. We defined individuals with mental health contact as those having had at least one consultation with a diagnosis code according to the mental health chapter of the ICPC-2 framework during the period (P01-P99). We also calculated the individual’s total number of GP consultations within the final year of the suicide. All indicators were generated by means of the date of death obtained from the Cause of Death Registry and the date of consultation obtained from the KUHR database.

For men and women in the general population, corresponding indicators as for suicide victims were calculated, using yearly population figures derived from the website of Statistics Norway as respective denominators [19]. We calculated yearly and monthly rates of contact with GPs in primary care throughout the period, before calculating the average contact rates during the period, relative to the mean population figure among men and women as respective denominators. For the population controls, we excluded suicide victims’ consultations in their respective year of death prior to the calculations.

Four age groups were generated according to age in the year of death for suicide victims, and according to age in the respective observation year for the population controls (i.e. 15–29, 30–44, 45–59, and 60 years and older).

### Statistical analysis

Prior to comparing rates of contact and frequency of consultations between suicide victims and the general population, we tested whether suicide victims’ rates of contact within a year of the suicide differed across the study period, using the Chi-square test for association. We then calculated rates of contact with GPs in primary care within a year of and within a month of the suicide, in addition to rates of mental health contact within the last year. We compared the rates of suicide victims with the average rates among the population controls by estimating relative risks (RR) with 95% confidence intervals (CI). Relative risks were estimated across all ages in addition to separately for each age group. We also tested for differences in frequency of contact within the last year between suicide victims and the population controls, by estimating the mean number of consultations with 95% confidence intervals. We considered the groups significantly different if no overlap in confidence intervals were present. Lastly, we estimated relative risks for consultations the last month by comparing the general population and suicide victims with and without prior mental health contact in primary care within the last year. All analyses were stratified by sex and conducted applying the Stata/SE version 15 software.

## Results

### Overall contact with GPs in primary care

Consultation rates with GPs in primary care within a year of the suicide were high and stable throughout the period, with no significant difference in rates of contact over years for neither males (χ^2^ 5.25; d*f* 8) nor females (χ^2^ 4.49; d*f* 8). In total, 82.4% of suicide victims consulted their GP within a year of the suicide. A significantly higher share of female (89.0%) than male suicide victims (79.6%) consulted GPs in primary care within the last year (RR 1.12; 95% CI 1.09–1.15). The sex difference among suicide victims were less pronounced than among the population controls, were 79.5% of females and 64.3% of males had consulted GPs in primary care within a year (RR 1.24; 95% CI 1.23–1.25). The consultation rates among suicide victims were significantly higher than the rates in the general population, evident for males (RR 1.24; 95% CI 1.22–1.26) and females (RR 1.12; 95% CI 1.10–1.14) alike (Table 1). In addition to higher overall rates of contact, suicides victims of both sexes consulted their GPs significantly more often than did their respective population controls. Among males, suicide victims consulted their GP on average 5.1 times (95% CI 4.9–5.3) within a year, compared to 2.4 times (95% CI 2.3–2.5) among the population controls. The equivalent rates among females were 7.7 consultations (95% CI 7.3–8.1) for suicide victims, compared to 3.4 consultations (95% CI 3.3–3.5) among the population controls.

**Table 1:** GP consultations 2006–2015

			Any consultation last year				Any consultation last month				Mental health consultation last year			
	Population	Suicides	Population	Suicides			Population	Suicides			Population	Suicides		
	N	N	%	%	RR	95% CI	%	%	RR	95% CI	%	%	RR	95% CI
Men														
All ages	1,976,598	3481	64.3	79.6	1.24	1.22–1.26	15.6	34.8	2.23	2.13–2.33	9.4	49.4	5.23	5.05–5.41
15–29	482,701	700	53.4	68.1	1.28	1.21–1.34	9.2	19.9	2.15	1.85–2.50	8.3	38.9	4.71	4.29–5.17
30–44	535,514	935	56.7	77.9	1.37	1.33–1.42	11.8	32.6	2.77	2.52–3.03	10.5	56.5	5.39	5.09–5.70
45–59	491,615	948	64.9	81.4	1.25	1.22–1.29	15.8	39.2	2.48	2.29–2.68	10.5	54.9	5.20	4.91–5.52
60+	466,768	898	83.7	88.5	1.06	1.03–1.08	26.4	43.9	1.66	1.54–1.79	8.3	44.3	5.33	4.95–5.74
Women														
All ages	1,999,634	1445	79.5	89.0	1.12	1.10–1.14	22.7	46.4	2.05	1.94–2.16	14.4	67.2	4.65	4.49–4.83
15–29	462,283	257	74.1	82.5	1.11	1.05–1.18	17.6	37.0	2.10	1.79–2.47	12.1	55.3	4.58	4.10–5.11
30–44	509,891	352	78.3	90.3	1.15	1.11–1.19	21.6	47.7	2.21	1.98–2.46	16.5	73.9	4.47	4.20–4.76
45–59	471,253	468	79.2	88.7	1.12	1.08–1.16	22.3	50.4	2.26	2.06–2.47	16.6	71.8	4.33	4.09–4.58
60+	556,207	368	85.4	92.7	1.09	1.05–1.12	28.1	46.5	1.65	1.48–1.85	12.7	63.3	4.99	4.62–5.40

Contact with GPs in primary care generally increased with age. This pattern was evident for suicide victims and the general population alike (Table 1). Across all age groups, suicide victims had significantly higher rates of contact within a year than had the population controls. This pattern was particularly evident for males, except for those of older age, where suicide victims (88.5%) had rates of contact more similar to males in the general population (83.7%). Although significantly different, the contact patterns among female suicide victims resembled more the patterns of their respective population controls. Among both suicide victims and the population controls, the overall lowest rates of contact were among those of younger age, in particular males, where 68.1% of suicide victims and only 53.4% of the population controls aged 15 to 29 years had consulted their GP within a year.

One month prior to suicide, 34.8% of male and 46.4% of female victims had consulted their GP. Both male (RR 2.23; 95% CI 2.13–2.33) and female suicide victims (RR 2.05; 95% CI 1.94–2.16) were more than twice as likely to consult their GP compared to the average monthly contact rate among the population controls (Table 1). Higher rates of contact among suicide victims than among the population controls were present across all age groups, with the least difference found among those of older age for both sexes. While monthly rates of contact generally increased with age among males, rates of contact were more stable across age among females. Among victims in contact with primary care the last month prior to suicide, 82.8% had previously consulted the same GP during their last year of life.

### Mental health contact with GPs in primary care

A considerable share of suicide victims had at least one mental health consultation within the last year prior to suicide. In total, 49.4% of male and 67.2% of female suicide victims had such a contact with GPs in primary care. The corresponding rates for males and females in the general population were no more than 9.4 and 14.4%, respectively (Table 1). Both male (RR 5.23; 95% CI 5.05–5.41) and female suicide victims (RR 4.65; 95% CI 4.49–4.83) were considerably more likely to have such a consultation within a year compared to the population controls. This pattern was evident across all age groups. For both sexes and for suicide victims and the general population alike, the highest rates of mental health contact were among the middle age groups. Among those between 30 and 59 years of age, more than half of male suicide victims and more than 70% of female suicide victims had such a consultation within a year of the suicide. As with consultations for any reason, younger males had the overall lowest rates of mental health contact, evident in only 38.9% of male suicide victims.

Within the last month prior to suicide, 21.4% of male and 32.5% of female suicide victims had mental health contact with a GP in primary care. Among all suicide victims consulting primary care during the last month, as many as 74.6% of males and 84.6% of females had at least one mental health contact with a GP at some point during their last year of life.

### Contact patterns the last month prior to suicide

Finally, we compared rates of contact during the last month prior to suicide between individuals with and without prior mental health contact with GPs in primary care within the last year. Using the general population without prior mental health contact as reference, the analyses, adjusted for age, revealed considerable differences in health care use the last month between those with and without such contact, evident for males and females alike (Table 2). Both male (RR 1.53; 95% CI 1.41–1.66) and female suicide victims (RR 1.45; 95% CI 1.27–1.67) without prior mental health contact the last year consulted GPs at a higher rate within the last month compared to individuals in the general population with a similar history. Furthermore, the average monthly contact rate among the population controls with prior mental health contact within the last year were well above that of suicide victims without such contact for both sexes. Both males (RR 2.63; 95% CI 2.61–2.65) and females (RR 2.08; 95% CI 2.07–2.10) among the population controls were significantly more likely to have a primary care consultation the last month than were their respective counterparts without prior mental health contact. Male suicide victims with prior mental health contact had an even higher likelihood of consulting their GP (RR 3.57; 95% CI 3.39–3.75), where as many as 49.6% consulted their GP within the last month prior to suicide. With a contact rate of 56.3%, female suicide victims with prior mental health contact had the overall highest rate of contact within a month of the suicide, and were considerably more likely to consult their GP than were females in the general population without such a history (RR 2.85; 95% CI 2.69–3.02).

**Table 2:** GP consultations last month 2006–2015

	Men				Women			
	N	% consultation	RR	95% CI	N	% consultation	RR	95% CI
Total	1,980,079	15.7			2,001,079	22.7		
Contact history last year								
No MH (Population)	1,799,201	13.7	1.00	Reference	1,719,831	19.7	1.00	Reference
No MH (Suicides)	1861	21.8	1.53	1.41–1.66	520	28.7	1.45	1.27–1.67
Prior MH (Population)	177,397	35.4	2.63	2.61–2.65	279,803	40.8	2.08	2.07–2.10
Prior MH (Suicides)	1620	49.6	3.57	3.39–3.75	925	56.3	2.85	2.69–3.02
Age category								
15–29	483,401	9.3	1.00	Reference	462,540	17.6	1.00	Reference
30–44	536,449	11.8	1.24	1.23–1.25	510,243	21.6	1.18	1.17–1.19
45–59	492,563	15.9	1.66	1.64–1.68	471,721	22.4	1.22	1.21–1.23
60+	467,666	26.4	2.85	2.82–2.88	556,575	28.1	1.59	1.58–1.60

*MH* Mental health consultation in primary care last year

## Discussion

This register-based study of the Norwegian population show that contact with GPs in primary care is common in the time leading up to suicide. The consultation rates among suicide victims were high and stable over the period, with approximately 80% of men and 90% of women consulting GPs in primary care within a year prior to the suicide. With rates of 34.8% for men and 46.4% for women within a month of the suicide, these rates are remarkably similar to those found in a recent review of health care use prior to suicide [4]. Contrary to previous studies including various control samples of non-suicides [3, 20], our findings revealed considerable differences in rates of health care use between suicide victims and the general population. Both one year and one month prior to suicide, for both sexes and across all ages, suicide victims consulted GPs in primary care at a significantly higher rate than did the general Norwegian population. Moreover, suicide victims had on average about twice the number of GP consultations within a year than had the general population.

The difference in rates of contact between suicide victims and the general population were even more pronounced when considering mental health consultations. About half of male suicide victims and about two thirds of female suicide victims had at least one mental health consultation within the last year prior to suicide, as compared to about 10 and 15% for males and females in the general population. Although mental disorders are argued to be at the core of the problem and found present in about 90% of suicides [21, 22], a considerable share of suicide victims in this national sample had no mental health contact with their GP within a year of the suicide. To what extent this is due to low levels of help-seeking, already ongoing treatment in the specialist health care services, or the inability to identify or correctly classify mental health problems within a primary health care setting, is difficult to delineate. Anyhow, the rates of mental health contact among suicide victims were manifold those of the general population for both sexes and across all ages.

Our study revealed considerable variation in rates of contact by age. Younger individuals had the overall lowest rates of contact with GPs in primary care, evident in both men and women, among suicide victims and the general population alike. As mental disorders is a significant risk factor for suicide in young adults [23, 24], the general low level of help-seeking behaviour in younger males remain a concern [25], indicating that alternative preventive measures will need to be taken in order to reach this group of the population who are less likely to consult any kind of health care service [5, 26]. Interestingly, although men in general have considerable lower rates of contact with the primary health care services than have women, male suicide victims displayed rates of contact more similar to those of women in the general population. As expected, due to increased morbidity, older individuals have high rates of contact with GPs in the primary health care services. However, the somewhat lower rate of mental health contact among those of older age is less expected and may be attributed to a perceived stigma towards seeking help for mental health problems among the elderly [5]. It may also be that actual mental health problems in some elderly goes undetected due to other pressing health complaints, for instance that conditions such as sleep disturbances or loss of appetite are interpreted as common somatic conditions and not as possible mental health issues.

Finally, a closer inspection of the rates of contact one month prior to suicide revealed some interesting patterns. The contact rates among suicide victims without prior mental health contact in primary care within the last year deviated only modestly from the corresponding group in the general population, evident for both sexes. On the other hand, the rates of contact among suicide victims with prior mental health contact were high this close to the suicide. About half of both male and female suicide victims with prior mental health contact consulted their GP within a month of the suicide. However, their contact patterns resembled more those of the general population with prior mental health contact than suicide victims without. As suicide among one’s patients is a rare event [3], and as suicide victims without any known mental health history differed only to a limited extent from the general population, perhaps greater optimism for prevention on behalf of GPs is warranted if one targets those with previous mental health contact in the primary or specialised health care services [11]. In accordance with findings of previous studies, these individuals appear to make contact closer in time to death and may thus be more reachable, for instance through referral to specialised mental health care.

### Strengths and limitations

A large sample size and registry-based data covering the entire Norwegian population over several years is a significant strength of the present study, and allows for a more detailed investigation into contact patterns across sex and age compared to previous studies. Despite major sex differences in contact with the health care services, only few previous studies have reported contact rates for men and women separately. Our study also include contact rates for the middle age groups, a group of the population largely neglected in the previous research literature. As private health care make up only a small proportion of the overall health care use in Norway, our national data on use of the primary health care services will cover most primary health care use during the period. Due to the referral based fee-for-service system, lack of registrations of actual help-seeking behaviour on behalf of GPs is not likely to pose a problem of any particular magnitude. Moreover, the Norwegian Cause of Death Registry has a near complete coverage, and the quality of data in the registry is considered good [15]. Thus, the possibility to investigate and contrast health care use between suicide victims and the general population, making use of high quality data, increases the reliability of the findings reported.

A limitation of the present study is that we did not have access to linking data on specialist psychiatric health care use. Inpatient psychiatric admission is a known risk factor for suicide, with individuals being at increased risk of suicide both during and shortly after discharge [27]. A study linking suicide death during the period from 2009 through 2011 to registrations in the Norwegian Patient Register found that altogether 36.8% of male and 54.6% of female suicide victims had been in contact with a psychiatric institution at some point during their last year of life, and furthermore that 4.6% of victims died while admitted to a psychiatric institution [28]. It is therefore unavoidable that some suicide victims in this study were receiving specialist psychiatric treatment at the time of death, and as such, had no need of consulting their GP within the last time prior to suicide. Nevertheless, a considerable proportion of suicide victims did have recent mental health contact with GPs in primary care, accounting for at least some degree of overlap across the different services. More research making it possible to link data and establish patient trajectories across primary care, inpatient and outpatient mental health care would give a more nuanced picture of how the overall health care use of suicide victims compares to that of the general population.

## Conclusions

For suicide prevention efforts to be effective, accurate identification of individuals at risk is required. As utilisation of health care services is universally accessible in Norway through the national health insurance, limited access to health care is not likely a critical issue for suicide prevention. Due to increased research-based knowledge, GPs may capitalise on the opportunity for prevention by being aware of clinical, age, and sex specific factors in assessing patients’ possible decisions to deliberately end their own lives [3]. A crucial prevention task of physicians, particularly those working in primary care settings, consist the recognition of a suicidal crisis in patients attending their practice. However, a recent meta-analysis found that suicidal intent is overtly communicated in only about half of cases [29]. The most promising interventions have therefore been argued to be physician education, means restriction, and gatekeeper education [2]. It has further been argued that the primary task remains the identification and treatment of mental disorders [30]. Despite the fact that most suicide victims were in contact with the primary health care services during the year prior to suicide, it may be that some did not receive adequate attention or appropriate treatment. The large number of individuals consulting primary health care in the time leading up to suicide thus highlight the primary care physician’s role in suicide prevention, for instance by referral of individuals at risk to specialist mental health care [3].

## Abbreviations

CI: Confidence interval
GP: General practitioner
ICD: International classification of diseases
ICPC: International classification of primary care
KUHR: Database for the reimbursement of health expenses
RR: Relative risk
